# 

**Published:** 1989-05

**Authors:** 


					r . .. wi BR323
* 1 04 <YO *">
^  y" J- 1989
I: .?;j,7~Z~7~SEO: B33740000
CICO~C!n*U!>?ICAL
BRISTOL
MEDICO
CHIRURGICAL
TOURNAL
?
. *
VOLUME 104(ii)
MAY 1989
SPECIAL ISSUE
CARCINOMA OF THE BREAST AND
THE NATIONAL BREAST
SCREENING PROGRAMME
also
PROSTHESES TO CONTROL
URINARY INCONTINENCE AND
COLOSTOMY INCONTINENCE
THE MEDICAL JOURNAL OF THE SOUTH WEST

				

